# Audio Described vs. Audiovisual Porn: Cortisol, Heart Rate and Engagement in Visually Impaired vs. Sighted Participants

**DOI:** 10.3389/fpsyg.2021.661452

**Published:** 2021-04-01

**Authors:** Ana M Rojo López, Marina Ramos Caro, Laura Espín López

**Affiliations:** ^1^Department of Translation and Interpreting, Faculty of Arts, University of Murcia, Murcia, Spain; ^2^Department of Human Anatomy and Psychobiology, Faculty of Psychology, University of Murcia, Murcia, Spain

**Keywords:** audio description, accessibility, visually impaired audiences, porn, engagement, sexual arousal, heart rate, cortisol

## Abstract

Audio description remains the cornerstone of accessibility for visually impaired audiences to all sorts of audiovisual content, including porn. Existing work points to the efficacy of audio description to guarantee immersion and emotional engagement, but evidence on its role in sexual arousal and engagement in porn is still scant. The present study takes on this challenge by comparing sighted and visually impaired participants’ experiences with porn in terms of their physiological response [i.e., cortisol and heart rate (HR)] and self-report measures of affect [Positive and Negative Affect Schedule (PANAS); Watson et al., 1988], anxiety [State-Trait Anxiety Inventory (STAI); Spielberger et al., 1970], sexual reactivity and arousal [Sexual Inhibition/Sexual Excitation Scale (SIS/SES); Moyano and Sierra (2014); and the Ratings of Sexual Arousal (RSA); Mosher (2011)], and narrative engagement or transportation [The Transport Narrative Questionnaire, Green and Brock (2013)]. 69 Spanish participants were allocated into three different groups: 25 sighted participants who watched and heard the porn scenes in their audio-visual version (AV); 22 sighted participants who listened to the audio described version without images (AD); and 22 visually impaired participants who also listened to the audio described version without images (ONCE). Overall, results on physiological and self-report measures revealed no significant differences between groups or different versions of the clips. The analysis of cortisol reactivity to porn as the maximum increase or decrease in cortisol (t+12) with respect to baseline values (t−20) revealed no significant differences between the groups, but pointed to a higher percentage of non-responders than responders in the three groups, the highest being found in the ONCE group. As for participants’ cardiac response to the clips, no significant differences were found across the groups, with the highest HR levels being registered in the baseline phase. Self-report measures revealed significant between-group differences in negative affect. The ONCE group displayed the highest pre-task levels of negative affect and was the only group that showed a decrease in negative affect after exposure to the clips. Sighted and visually impaired participants reported to be moderately aroused and immersed in the films, regardless of exposure to AV or AD porn. In addition, correlations found between participants’ levels of self-report sexual arousal and transportation and post-task affect pointed to a positive relationship between exposure to porn and perceived levels of sexual arousal and affect. Results from the study reflected the efficacy of audio description in providing sighted and visually impaired audiences with a similar experience to that offered by original AV porn scenes. This study is exploratory but provides valid, initial groundwork for further research on the impact of audio description on porn reception.

## Introduction

Accessibility is one of the major challenges of the 21st Century. The quest for a more global and inclusive world has brought about the desire to make products, services and even entertainment accessible to all people, regardless of their abilities. Books have been recorded in audio for those with vision loss, for lovers of multi-tasking, or even to combat loneliness or commuting boredom. Film dialogs have been made readable in subtitles for those with hearing loss or language proficiency issues, and film images have been audio described for visually impaired audiences. And yet, the best efforts do not seem to be enough. Focusing on visual impairment, figures show that more than 30 million blind and partially sighted citizens in Europe are estimated to have poor access to audiovisual media services such as broadcast television, TV equipment related to digital television services, e-books, and e-commerce relevant for purchasing tickets for cultural events (European Accessibility Act, 2018).

The American Council of the Blind defines Audio Description (hereafter, AD) as a narration service provided–at no additional charge to the user–to guarantee the accessibility of the visual images of theater, television, films, and other art forms for visually impaired people. AD is added to the soundtrack of a program during existing pauses in dialog and describes “what the sighted person takes for granted–those images that a person who is blind or visually impaired formerly could only experience through the whispered asides from a sighted companion.” (From The Audio Description Project, An Initiative of the American Council of the Blind, Available at https://acb.org/adp/ad.html).

The image of the “whispering asides from a sighted companion” illustrates rather well the difficulties of making porn accessible to the visually impaired community. Watching porn is an intimate and erotic experience usually shared only with one’s partner. Porn consumption involves a huge amount of shame for a number of different religious, cultural or ideological reasons. Many of those are related to sex in general, but some may derive from feminist beliefs in the anti-women essence of porn or from the poor reputation of the porn star culture. But watching porn is not necessarily shameful or harmful; voices are also raised in favor of porn and its beneficial effects for both men’s and women’s understanding of their sexual desires and construction of their identities (McCormack and Wignall, 2017; Daskalopoulou and Zanette, 2020) or even for their distraction from loneliness, distress, boredom or even pandemic-related negative emotions (Grubbs, 2020; Mestre-Bach et al., 2020). Even if porn gets stigmatized by society, billions of people consume it every year. The adult films industry makes almost 100 billion dollars a year. According to the 2019 annual report by the Internet site Pornhub, this site received 42 billion visits during the year, which means there was an average of 115 million daily visits (average age = 36; 32% of women and 68% male). Interestingly, figures increased exponentially during the COVID-19 pandemic and the national measures of self-isolation and quarantine, with a peak increase of 24.4% on March 25, 2020 after Free Pornhub Premium was offered to encourage people to stay indoors (Pornhub’s coronavirus update, April 14, 2020).

Further than the existing debate on its good or bad consequences (Zimbardo, 2016), watching porn–at least the “ethical” type that complies with legal regulations–is now legal in most countries, and as such it should be made accessible to all, including visually impaired citizens. As Snyder (2016), director of The Audio Description Project at the American Council of the Blind puts it, “people who are blind have every right to access porn as they do classical Shakespeare or any other kind of video.” There are also good economic reasons for developing porn for the blind, since “(T)he blind community is large and has buying power” (Snyder, 2016). In the United States, some projects have been developed to make porn accessible for the blind. In 2006, the website Pornfortheblind.org was conceived as a library of MP3 files with descriptions of popular adult videos recorded by volunteers. The site managed to attract 150,000 visitors per month. Although this platform no longer exists, it highlighted the immense relevance and impact of the initiative. More recently, in 2016, the giant Pornhub presented the initiative Described Videos, which offers a selection of their top performing films with added AD for their visually impaired users. The initiative is also promoted by the renaissance of audio porn with new platforms offering erotic audio stories (e.g., Dipsea), described sex films, and not safe for work (NSFW) podcasts.

Once porn is made accessible, the question arises as to what point the experience provided by AD porn is similar to that of viewed porn. The present study explores this question by comparing the experiences of sighted vs. partially sighted and blind participants in terms of their physiological response [i.e., cortisol and heart rate (HR)] and self-report measures of sexual arousal, emotional and narrative engagement. The relevant theoretical background of the study includes a brief review of relevant studies on AD, emotions and sexual arousal.

In translation studies, AD has been considered a mode of intersemiotic translation in which the images of audiovisual texts (e.g., films, theater, and documentaries) are translated into words. It is, thus, usually researched as a form of audiovisual translation (AVT henceforth). The current focus on accessibility and the desire to regulate the profession of the audio describer has placed AD in the spotlight of translation research. Most studies are descriptive and focus on the analysis of existing scripts (Jiménez, 2010) or the creation of guidelines (Rai et al., 2010), but experimental research on the processes involved in the creation and reception of AD has gained momentum in the last decade. The majority of experiments and quasi-experiments have focused almost exclusively on the selection of the relevant information to audio describe, either through the analysis of verbal descriptions from sighted participants (Mazur and Kruger, 2012) or through the use of eye-trackers that allow researchers to identify where sighted viewers focus their visual attention (Orero and Vilaró, 2012).

More recently, the importance of the psychological factors that may influence the creation and reception of AD has been highlighted by a number of studies focused on emotions (Ramos and Rojo, 2014; Ramos, 2015, 2016) and their link to other phenomena highly related to fictional emotions, such as immersion (Wilken and Kruger, 2016; Walczak, 2017) and presence (Fryer and Freeman, 2013; Fryer and Jonathan, 2014; Walczak and Fryer, 2018). Studies on emotion in AD are particularly relevant for the present study given the close connection between emotional and sexual reactions. The definition of sexual desire as an emotion is not exempt of problems. Differences with the emotion of romantic love relegate sexual desire to the category of a biological surge closer to hunger or thirst than love. And yet, when the basic components of an emotion are considered, sexual desire appears as a most typical one. Sexual arousal involves both physiological activation and subjective appraisal; moreover, it comprises feelings of pleasure and enjoyment with features of arousal and valence (Walter et al., 2008). Regardless of whether or not sexual arousal can be considered an emotion, their interaction is obvious. Sexual and emotional activation frequently influence one another: emotional states can condition or predict sexual arousal (Peterson and Janssen, 2007; Nobre and Pinto-Gouveia, 2008) and sexual arousal is capable of influencing or even inhibiting certain emotional reactions such as disgust (Borg and de Jong, 2012).

Most of the AD studies on emotion are based solely on self-report measures. For instance, the study by Fryer and Freeman (2013) compared the impact of sound effects and verbal pictures on the audience’s presence–where presence is understood as the sense of connection of users of media technologies with real or fictional environments and the objects and people in them [The Independent Television Commission–Sense of Presence Inventory (ITC–SoPI), Lessiter et al., 2001]. The study measured four dimensions of presence (i.e., spatial presence, ecological validity, engagement, and negative effects) among three groups of participants (blind, partially sighted and sighted) exposed to a film sequence in three different conditions: without AD, with “standard” AD; and with AD specifically designed to convey the cinematic medium of the film, including camerawork (e.g., close-up, tracking) and editing (e.g., cuts and dissolves). Their results suggested that presence and ecological validity were enhanced with the cinematic version of the AD, whereas engagement depended largely on audience comprehension. Interestingly, levels of presence were higher in the visually impaired than in the sighted audience. Another example was the work by Fryer and Jonathan (2014), who explored the link between emotion and presence in visually impaired participants when listening to two different AD versions (i.e., one using text-to-speech technology and the other with human voice) in film clips eliciting different emotions (i.e., fear and sadness). Results showed that neither participants’ levels of presence (also measured by the ITC–SoPI) nor their emotional reactions (EES, Gross and Levenson, 1995) or empathy (Dillon, 2006) were affected by the different versions of the AD or by the different emotions.

There are also some AD studies that combine both subjective and physiological measurement. Fryer (2013), for instance, used physiological measures–i.e., electrodermal activity (EDA), HR, and heart rate variability (HRV)–and self-report questionnaires to test whether the semantic information provided by AD reduced the emotional experience and sense of presence (also measured by the ITC–SoPI inventory) fostered by music and sound effects in three film clips eliciting fear and three eliciting sadness. Results differed between emotion categories (sadness/fear) but showed that AD did not lead to a reduction in presence or in levels of elicited emotion. Of all physiological measures, HRV was the only measure to show a significant response.

Ramos (2015, 2016) also explored AD emotional reception by means of questionnaires and by measuring HR in visually impaired and sighted participants. Her studies compared the emotional power of audiovisual texts with and without AD for the emotions of fear, sadness and disgust, and assessed the efficacy of different AD styles: a more descriptive and neutral AD vs. a more subjective and narrative one. Results suggested that the emotional response triggered by AD could be as strong as the one evoked by the original scenes, especially for the emotions of fear and sadness. The inclusion of subjective information in the AD was also widely accepted by both sighted and partially sighted audiences, although differences were found between the different emotional scenes. Ramos and Rojo (2014) also provided results pointing to the role of film typology in the emotional reception of AD. Differences in the emotional response of sighted vs. partially sighted and blind participants were more prominent in avant-garde than in narrative films.

Relatedly, Iturregui-Gallardo (2019) studied the emotional experience of blind, partially sighted and sighted audiences, but used Audio Subtitles (AST)–i.e., subtitles read aloud for those who cannot see them–instead of AD. According to the author, AST were the perfect intersection between subtitles, AD and voice-over. The study analyzed the emotional reception of two AST strategies: dubbing effect and voice-over effect. The audience’s emotional response was measured by using EDA, HR, and self-report instruments. Self-report data showed that AST were more emotionally activating when used with dubbing than with voice-over effect, but only for the emotion of fear. Questionnaires also revealed similar levels of emotional activation in blind, partially sighted and sighted audiences when exposed to the same stimuli. However, results from physiological measures were not conclusive.

Existing results point to the fact that AD is capable of eliciting powerful emotional reactions, which are generally as strong as the ones triggered by the original audiovisual scenes. Nevertheless, results seem to depend largely on the typology of the films and emotions used as stimuli, as well as on the measurement methods employed. The present study investigates the differences in the experiences of sighted vs. visually impaired participants when watching or listening to AD porn films. The reception of AD porn and its physiological and psychological effects on the audience is still an unexplored field that deserves further attention to ensure equal access to porn for everyone.

The study compared sighted and visually impaired participants’ levels of physiological reactivity and self-reported sexual, emotional and narrative engagement when exposed to two different versions of porn scenes: an audiovisual version, including image and sounds (AV) and an audio version, including the AD and the original soundtrack (AD).

Two main hypotheses were posed to test the effect of AD porn (H1 and H2):

(1) No differences were expected between AD and AV porn regarding participants’ levels of physiological reactivity and self-reported sexual, emotional and narrative engagement.
(2) No differences were expected either between sighted and visually impaired participants’ levels of physiological reactivity and self-reported sexual, emotional and narrative engagement when exposed to the AD version without images.

Two sub-hypotheses were also posed regarding the expected effect of porn (H3) and the correlation between the different measures (H4):

(3) Exposure to porn stimuli was expected to increase participants’ levels of physiological reactivity and sexual, emotional and narrative engagement. In general, we expected:

(3.1) Increased sexual arousal would be indicated by an increase in HR indices, a decrease in cortisol secretion and high self-report scores on sexual arousal.
(3.2) Increased emotional engagement would be indicated by higher levels of self-reported positive affect and lower levels of self-reported anxiety.
(3.3) High narrative engagement would be indicated by high self-report scores on transportation.

(4) Self-report measures of sexual arousal were expected to correlate positively with HR and scores on transportation and positive affect, but negatively with cortisol and anxiety levels.

## Materials and Methods

### Participants

Sixty-nine Spanish participants took part in the experiment. All sighted participants were recruited at the University of Murcia (Spain), whereas all visually impaired participants were contacted through the National Organization for the Blind (ONCE) at its headquarters in the city of Murcia. The ONCE gave permission to conduct the experiment and searched for volunteers among their visually impaired members. All participants granted their consent according to the Declaration of Helsinki, and the protocols were approved by the University of Murcia Ethics Committee. Participants were informed of the general purpose of the study and were told that they could leave the experiment at any point.

Information on the general characteristics of the sample was collected prior to the experiment (see Table 1 below). Data were also collected on health factors likely to influence participants’ physiological response, such as the day of the menstrual cycle (Hamidovic et al., 2020). This information was nevertheless not included in our analyses, since most participants admitted not being sure of their answers.

**TABLE 1:** General sample characteristics.

**Variables**		**AV (*N* = 25)**	**AD (*N* = 22)**	**ONCE (*N* = 22)**
Socio- economic status	High	4%(1)		
	Average	84%(21)	100%(22)	77.3%(17)
	Low	12%(3)		22.7%(5)
Sexual orientation	Heterosexual	72%(18)	72.72%(16)	86.4%(19)
	Bisexual	24%(6)	18.2%(4)	4.54%(1)
	Homosexual	4%(1)	4.54%(1)	4.54%(1)
	Pansexual		4.54%(1)	
	Asexual			4.54%(1)
Education level	Basic			31.8%(7)
	High school	68%(17)	63.6%(14)	18.2%(4)
	University studies	32%(8)	31.7%(7)	45.5%(10)
	Others		4.5%(1)	4.5%(1)

The sample was composed only by women aged 18–42 years old, with a mean age of 23.98. The age range was established under the assumption that young audiences would feel more comfortable watching explicit sex scenes. All blind participants met the criteria set by the ONCE^1^ to be considered visually impaired, three of them were totally blind, and the rest were partially blind.

Participants were allocated into three different experimental groups:

– UM_AV: 25 students from the University of Murcia watched and heard the scenes in their audiovisual version.
– UM_AD: 22 students from the University of Murcia listened to the audio described version without images (original audio + AD, black screen).
– ONCE: 22 visually impaired participants listened to the audio described version (original audio + AD, black screen).

### Materials and Stimuli

Two porn scenes were selected as stimuli for the experiment. They had been previously validated for sexual activation in heterosexual women and obtained comparable results in terms of sexual arousal (Gómez-Lugo et al., 2016). They lasted 6 min each and had a similar content. They both showed a heterosexual encounter between a woman and a man and depicted similar sexual practices (foreplay, cunnilingus, fellatio, and intercourse). The two sexual encounters were initiated by the woman and focused mainly on her pleasure. Before the explicit content, there was a context of emotional development between the actors, and actors’ expressions and appearance were natural (cf. Gómez-Lugo et al., 2016). Clips were treated as one 12-min video in order to give enough time for the physiological reactions to unveil. The two scenes were presented in randomized order: half of the participants in each group watched or heard scene 1 first and then scene 2, whereas the other half watched or heard scene 2 first and then scene 1.

A team of professional audio describers created and recorded the audio description of the scenes. Although some studies have claimed that intonation plays an important role in the reception of AD (Iglesias et al., 2011, 2015), the AD was recorded with a neutral intonation, following present recommendations in Spain (AENOR, 2005). As already stated, each scene was presented in two different versions: an AV version, including image and sounds, and an AD version, including the AD and the original soundtrack, but no images. The UM_AV group of sighted participants was exposed to the AV version of the clip, whereas the other two groups were exposed to the AD version in order to compare the experiences of visually impaired participants used to AD films (i.e., the ONCE group) and that of sighted participants (i.e., the UM_AD group) unaccustomed to AD.

### Instruments

Five self-report questionnaires were used to measure the participants’ subjective feeling component of emotion [i.e., the Positive and Negative Affect Schedule (PANAS), Watson et al. (1988); the State-Trait Anxiety Inventory (STAI), Spielberger et al. (1970)], sexual reactivity and arousal [the Sexual Inhibition/Sexual Excitation Scale (SIS/SES), Moyano and Sierra (2014); the Ratings of Sexual Arousal (RSA), Mosher (2011)] and their levels of narrative engagement or transportation [The Transport Narrative Questionnaire, Green and Brock (2013)].

The three questionnaires on emotional aspects and sexual reactivity were used to measure participants’ levels of affect and sexual reactivity before the experimental task. The PANAS (Watson et al., 1988) asked participants to rate the intensity of their affective state on a five-point Likert scale. The STAI (Spielberger et al., 1970) measured participants’ levels of state anxiety (STAI-S) and trait anxiety (STAI-T) on a four-point Likert scale. Lastly, the SIS/SES (in its short form), first developed in English by Carpenter et al. (2010), and later validated in Spanish by Moyano and Sierra (2014), consisted of 14 items evaluated on a four-point Likert scale. The 14 items were grouped into three subscales corresponding with different dimensions of sexual reactivity:

– SES (arousal): sexual arousal derived from social interactions (items 1, 3, 8, 10, 11, and 14).
– SIS1 (inhibition 1): the fear to lose sexual arousal due to a distraction or not being fully focused on sexual sensations (items 4, 9, 12, and 13).
– SIS2 (inhibition 2): the threat to lose sexual arousal if there is a risk of being surprised during sexual interaction or acquiring a sexually transmitted disease (items 2, 5, 6, and 7).

After film exposure, they completed again the PANAS and the STAI state, together with the RSA [Mosher (2011); validated in Spanish by Sierra et al. (2017)] and the Transport Narrative Questionnaire (Green and Brock, 2013). The RSA presented a seven-point Likert scale composed of five items: overall estimation of sexual arousal, estimation of the intensity of genital sensations, estimation of the sensation experienced, estimation of non-genital physical sensations and estimation of the level of self-absorption experienced in the sexual situation. A higher score indicated a higher level of sexual arousal. The Transport Narrative Questionnaire (Green and Brock, 2013) measured feelings of transportation or “the experience of cognitive, affective and imagery involvement in a narrative” (Green, 2004). The test consisted of a seven-point Likert scale with 12 items grouped into five subscales: cognitive attention (items 6 and 8), feeling of suspense (items 4, 5, and 9), mental imagery (items 1, 3, and 12), lack of awareness of surroundings (item 2), and emotional involvement (items 7, 10, and 11).

Heart rate and cortisol responses to the porn stimuli were also measured as indicators of physiological reactivity during sexual arousal and emotional engagement. Measuring the cortisol response has proven particularly useful in sexual arousal studies as a method to discriminate between sexual excitement and the stress response. The sympathetic nervous system (SNS) is activated in both situations, but cortisol is only active in the latter. Existing evidence on women’s physiological responses to erotic films shows that their cortisol levels generally decrease in response to sexual stimuli, a reaction associated with sexual desire and genital arousal (Hamilton et al., 2008). Nevertheless, there are intervening factors that may reverse the pattern, increasing cortisol secretion during sexual arousal, such as a history of childhood sexual abuse (Rellini et al., 2009) or even lower sexual satisfaction (Hamilton et al., 2008). These results suggest that cortisol may play a role in emotional responses associated with sexual anxiety and lower sexual functioning.

In our study, salivary cortisol was collected using the Salivette^®^ sampling device (Sarstedt, Nümbrecht, Germany). Four saliva samples were collected at four different points in time with reference to the start of the experimental task (sample t0): baseline t−20 (20 min. before the start of the experimental task), sample t0 (the start of the experimental task), sample t+12 (12 min. after the start of the task), and sample t+27 (27 min. after the start of the task). Participants were instructed to place the cotton swab inside their mouths for 2 min, not to chew the cotton because it may affect salivary protein composition as well as the flow rate (Bosch et al., 2011), and move the swab around in a circular pattern to collect saliva from all the salivary glands (Rohleder and Nater, 2009). The uncentrifuged saliva samples were stored at −80°C immediately upon collection, until analyses were performed. To reduce sources of variability, all four samples taken from each participant were analyzed in the same assay. The samples were analyzed by a competitive solid phase radioimmunoassay (tube coated), using the commercial kit Coat-A-Count Court (DPC, Siemens Medical Solutions Diagnostics). Assay sensitivity was 0.5 ng/ml. Cortisol levels were expressed in nmol/l, with coefficients of intra- and inter-assay variations of less than 10%.

Since cortisol response to activating stimuli reaches a maximum level within 15 min after treatment, the increase in cortisol during this time interval is most likely to warrant an optimum measure of stimuli responsiveness. Differences in cortisol values between baseline and t+12 values were used to measure responsiveness. A distinction was made between individuals who showed cortisol responses (responders) and those who did not (non-responders) within each group. Participants who did not show an increase in cortisol levels of 1.5 nmol/l above baseline after exposure to the film were considered as non-responders.

Mean HR (beats/min) was also measured in our study as an indicator of SNS activation. The SNS interacts with the adrenal cortex to maintain cardiovascular and metabolic homeostasis, and also plays a decisive role in the stress response and in sexual arousal. Even if HRV–usually measured as the standard deviation of normal to normal R-R (NN) intervals (SDNN) or the root mean square of differences of successive heartbeat intervals (RMSSD)–is claimed to be a more powerful indicator of modulation of the SNS in response to cognitive or emotional functions (Task Force of the European Society of Cardiology and the North American Society of Pacing Electrophysiology, 1996), HR may be a valid indicator in conjunction with other additional measures of SNS activation, such as cortisol secretion. Moreover, HR is non-invasive, and relatively easy to measure with a HR monitor. In the present study, participants’ HR was recorded with the Polar Vantage M HR monitor, a multisport watch with Polar Precision Prime sensor fusion technology. The Polar monitor allows the researcher to sample HR every 5, 10, 15, or 60 s (i.e., the interbeat interval at the specified epoch is recorded). For this study, the Polar monitor was programmed to sample HR values every 5 s following previous evidence on the validity of this reading to provide a valid measure of HR during laboratory tasks (Goodie et al., 2000). The KubiosHRV^®^ Premium Polar Software V3.2 (Biomedical Signal Analysis Group, University of Kuopio, Finland) was used to filter and analyze the signal obtained from the Polar records.

### Procedure

In order to accommodate the different audiences, two different experimental sites were used: the headquarters of the Spanish National Organization for the Blind (ONCE) in Murcia, and the Faculty of Humanities at the University of Murcia. In both cases, a quiet room isolated from external noise with a table and two comfortable chairs was reserved. The films were played in a 13″ MacBook Pro and through Sennheiser HD 219-S high-end headphones. Participants adjusted the volume of the headphones before the experiment would start. Each participant was received individually and informed of the need to wear a wrist HR monitor watch in order to record information about their HR when taking part in the experiment. They were also told that they would watch or listen to some porn clips and answer a series of questions, but the specific purpose of the study remained unknown to them.

Once their consent was given, they were assisted to adjust the watch to their wrist size. Levels of HR were measured continuously throughout the entire experimental session. The protocol started with a baseline phase of 20 min to allow participants to adapt to the experimental setting. During this phase, they remained seated, and baseline measures were obtained for HR. Baseline levels of cortisol were also measured and the STAI-T and STAI-S, PANAS, and SIS/SES were completed by the sighted. In the case of the visually impaired participants, these questionnaires were read aloud and filled-in by one of the experimenters.

Levels of cortisol in saliva were again measured right before film exposure. After these initial measurements, the researcher pressed the lap button on the HR monitor and the experimental task would begin. Participants were asked to relax and were left alone while they watched or heard the clips. Following the recommendation presented by the Task Force of the European Society of Cardiology and the North American Society of Pacing Electrophysiology (1996), only the middle 5 min of each phase were analyzed. Therefore, after eliminating the artifacts, the HR means for each 5-min phase (Baseline, task and recovery) were computed. HR was recorded in real-time and expressed as beats per minute (bpm).

**FIGURE 1:**
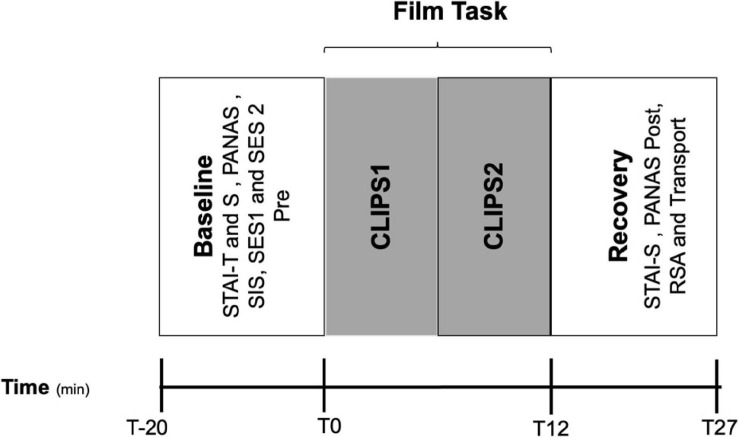
Different phases of the experimental protocol. Sequential salivary cortisol sampling (t-20 to t27).

After film exposure, the researcher pressed again the lap button on the HR monitor and salivary cortisol was also measured. Post-stimulus questionnaires were completed (PANAS and STAI-S, RSA, and Transport Narrative Questionnaire). Before leaving the room, cortisol was collected for the last time. The average duration time for the whole experiment was approximately 1 h (see Figure 1).

### Data Analysis

Cortisol and HR levels were tested for normal distribution and homogeneity of variance using the Kolmogorov–Smirnov and Shapiro–Wilk tests before the statistical procedures were applied. Of these analyses, only cortisol data revealed significant deviations from normality and were sqrt-transformed to approach a normal distribution. For an easy interpretation of the figures, the values in these represent raw values and not transformed values, they are mean ± standard error of mean (SEM).

One-way ANOVAs were conducted in order to test group differences (AV vs. AD vs. ONCE) on age, Body Mass Index (BMI), STAI-T, the SIS/SES, the RSA Scale and The Transport Narrative questionnaire and cortisol reactivity.

To assess group differences in STAI-S and PANAS-N and PANAS-P before and after the task, as well as cortisol and cardiac levels during the different phases of the protocol, we conducted separate repeated measures analyses of variance (ANCOVAs) with group as a between-subjects factor (AV vs. AD vs. ONCE) and time as a within-subjects factor (three times for HR: baseline, task and recovery phases; four times for cortisol (t−20; t0, t+12, and t+27; and two times for anxiety and for positive and negative affect: pre- and post-task).

Pearson’s correlations were calculated to assess whether participants’ variations in physiological reactivity, anxiety and affect measures were related to their scores on sexual arousal (RSA) and the transportation questionnaire.

Since significant differences in age, BMI and STAI-T levels were found between the groups (see the “Results” section below), these variables were introduced in the analyses as covariates for all measures, and *post hoc* contrast analyses were conducted. All results were corrected using the Greenhouse–Geisser procedure, where appropriate. *Post hoc* comparisons were performed using Bonferroni adjustments for multiple comparisons. As a measurement of the effect size, we report Partial Eta Squared (η^2^_*p*_) values. All statistical analyses were performed on SPSS 27.0.

## Results

### Anthropometric and Demographic Variables

Significant differences were found between the groups on age, BMI and STAI-T levels. The ONCE group was significantly older, showed higher BMI, and lower trait anxiety than the other groups. However, the groups showed similar scores in the 3 subscales corresponding with different dimensions of sexual reactivity. The main characteristics of the groups are shown in Table 2.

**TABLE 2:** Statistics for anthropometric and demographic variables for the full sample (AV = 25; AD = 22; ONCE = 22).

**Variables**	**Group**	**Minimum**	**Maximum**	**Mean**	**S.E.M**	***F***	***p*-value**
Age	AV	20	23	20.88	0.13	15.38	<0.001
	AD	19	42	21.81	1.49		
	ONCE	18	40	29.95	1.72		
BMI	AV	17.58	30.80	21.75	0.66	3.26	0.044
	AD	17.01	25.71	21.64	0.56		
	ONCE	16.87	19.52	23.66	0.60		
STAI Trait	AV	9	47	30.28	2.20	4.64	0.013
	AD	18	46	31.77	1.71		
	ONCE	11	39	24.09	1.44		
SES	AV	8	22	14.88	0.58	1.76	0.180
	AD	6	18	13.27	0.72		
	ONCE	11	20	14.40	0.55		
SIS1	AV	9	20	14.40	0.58	0.29	0.749
	AD	9	19	13.90	0.50		
	ONCE	7	21	14.50	0.68		
SIS2	AV	9	16	13.00	0.38	0.61	0.543
	AD	7	16	13.50	0.51		
	ONCE	8	16	12.77	0.50		

*BMI, body mass index; SEM, standard error of the mean; SES, sexual excitation scale; SIS, sexual inhibition scale.*

### Salivary Cortisol

**FIGURE 2:**
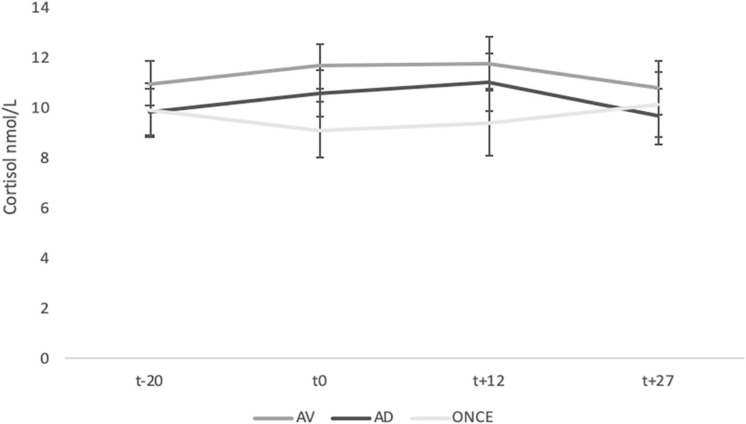
Means of salivary cortisol levels at the different phases of the experimental protocol for each group. Error bars represent standard error of the mean (SEM).

A repeated-measures ANOVA was conducted with time (4) as within-subject factor and group (3) as between-subject factors. The results did not show a significant main effect for the time factor [*F*(3; 189) = 1.07, *p* = 0.36, η^2^_*p*_ = 0.01] group [*F*(2; 63) = 0.43, *p* = 0.65, η^2^_*p*_ = 0.01] nor the interaction time × group [*F*(6; 189) = 0.90, *p* = 0.49, η^2^_*p*_ = 0.02] (see Figure 2).

As previously outlined, cortisol reactivity to porn was also analyzed as the maximum increase or decrease in cortisol (t+12) with respect to baseline values (t−20). Even if there are no clear guidelines for determining a definite cortisol response to distinguish between responders and non-responders, the fixed threshold classification criterion of 1.5 nmol/l baseline-to-peak-increase (Glienke and Piefke, 2017) was adopted in the present study. The univariate ANOVA revealed no significant differences between the groups [*F*(2; 66) = 1.69, *p* = 0.19, η^2^_*p*_ = 0.04], but the analysis pointed to a higher percentage of non-responders in the three groups, the highest being found in the ONCE group: AV 68% (*N* = 16); AD 68.2% (*N* = 14); ONCE 81.8% (*N* = 18) (see Table 3).

**TABLE 3:** Cortisol levels (nmol/L) in the three groups.

**Cortisol levels (nmol/L)**	**AV (*N* = 25)**	**AD (*N* = 22)**	**ONCE (*N* = 22)**	**Total sample**
T−20	11.58 (4.27)	10,69 (3.74)	9.47 (3.83)	10.62 (4.00)
T+12	12.43 (5.96)	11.67 (5.38)	8.57 (2.78)	10.96 (5.16)
Cortisol reactivity*	0.85 (3.15)	0.98 (5.18)	−0.89 (2.69)	0.33 (3.84)
% Cortisol responders/non-responders**	32% (*n* = 9)/68% (*n* = 16)	31.8% (*n* = 8)/68.2% (*n* = 14)	18.2% (*n* = 4)/81.8% (*n* = 18)	30.4% (*n* = 21)/69.6% (*n* = 48)

*Maximum increase in cortisol with respect to the baseline cortisol (t+12 to t−20). **% Cortisol responders taking as fixed threshold classification criteria a 1.5-nmol/l. baseline-to-peak increase. The values represent means and standard deviation of the mean in parenthesis.

### Cardiovascular Activity

**FIGURE 3:**
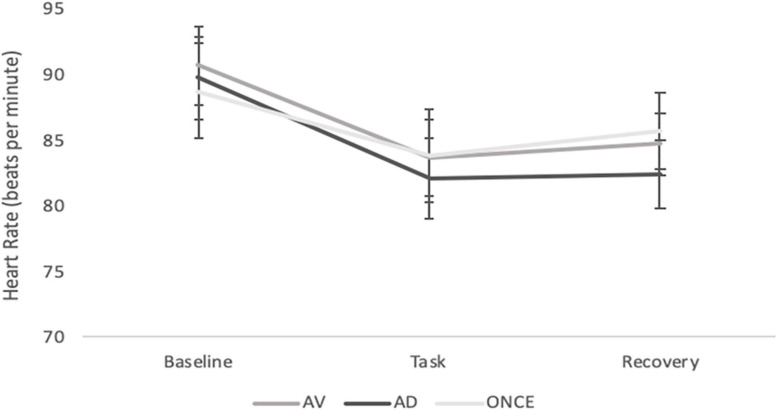
Estimated means of heart rate for each phase of the experimental protocol (Baseline, Task and Recovery). Error bars represent standard error of the mean (SEM).

Heart rate results did not report a significant main effect of the time factor [*F*(2; 126) = 0.21, *p* = 0.80, η^2^_*p*_ = 0.003] group [*F*(2; 63 = 0.04, *p* = 0.95, η^2^_*p*_ = 0.001] nor the time × group interaction [*F*(4; 126) = 0.96, *p* = 0.42, η^2^_*p*_ = 0.003] (see Figure 3).

### Subjective Measures

Results on the STAI-S did not show a significant main effect of the time factor [*F*(1; 63) = 0.24, *p* = 0.62, η^2^_*p*_ = 0.004], group [*F*(2; 63 = 0.34, *p* = 0.71, η^2^_*p*_ = 0.01] nor the time × group interaction [*F*(2; 63) = 1.58, *p* = 0.21, η^2^_*p*_ = 0.04].

Results on the PANAS-P did not show a significant main effect of the time factor [*F*(1; 63) = 0.44, *p* = 0.50, η^2^_*p*_ = 0.007], group [*F*(2; 63 = 1.37, *p* = 0.26, η^2^_*p*_ = 0.04] nor the time × group interaction [*F*(2; 63) = 2.14, *p* = 0.12, η^2^_*p*_ = 0.06].

Results on the PANAS-N did not show a significant main effect of the time factor [*F*(1; 63) = 1.77, *p* = 0.18, η^2^_*p*_ = 0.02]. However, a significant main effect of the group factor [*F*(2; 63 = 3.55, *p* = 0.03, η^2^_*p*_ = 0.10] and a marginal effect of the time × group interaction [*F*(2; 63) = 2.54, *p* = 0.08, η^2^_*p*_ = 0.07] were reported.

**FIGURE 4:**
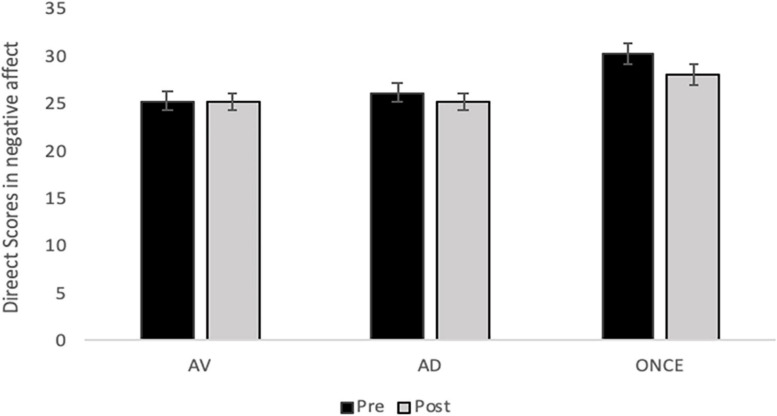
Estimated Means of Negative Affect for each phase (Pre and Post-Task) across the three groups. Error bars represent standard error of the mean (SEM).

*Post hoc* analyses revealed that the ONCE group showed the highest negative affect when compared with the other two groups (ONCE vs. AV *p* = 0.04; ONCE vs. AD *p* = 0.07). Significant between-group differences appeared only pre-task, with the ONCE group showing the highest levels of negative affect (ONCE vs. AV *p* = 0.009; ONCE vs. AD *p* = 0.03). The ONCE was the only group which showed a significant decrease in their negative affect after the experimental task (pre vs. post task *p* = 0.002). No significant pre-post task differences were reported for the other two groups (AV: pre vs. post task *p* = 0.94; AD: pre vs. post task *p* = 0.11) (see Figure 4).

Finally, scores on the RSA and total scores on the Transport Narrative questionnaire did not reveal significant differences between the groups, [*F*(2; 63) = 1.25, *p* = 0.29, η^2^_*p*_ = 0.03] and [*F*(2; 63) = 1.04, *p* = 0.359, η^2^_*p*_ = 0.03], respectively. Scores on the RSA for the three groups were: (ONCE *M* = 21.62 ED = 1.71); (AV *M* = 17.72 ED = 1.43); (AD *M* = 19.05 ED = 1.47). Total scores on the Transport Narrative questionnaire for the three groups were: (ONCE *M* = 49.17 ED = 2.85); (AV *M* = 43.24 ED = 2.39); (AD *M* = 45.14 ED = 2.45).

### Relationship Between Ratings of Sexual Arousal, Transportation and Physiological and Psychological Measures

The correlation analysis did not show an association between participants’ levels of subjective sexual arousal and transportation and their levels of HR and cortisol released during exposure to the porn clips.

However, a significant positive correlation was reported between their scores on the RSA and on the Transport Narrative questionnaire for the total score (Total) and its five subscales: cognitive attention (CA), feeling of suspense (FS), mental imagery (MI), lack of awareness of surroundings (LA), and emotional involvement (EI). A significant positive correlation was also reported between participants’ scores on the RSA and on the PANAS-P after exposure to the porn clips.

In addition, their total score on the Transport Narrative questionnaire and their scores for two of its subscales (i.e., cognitive attention, mental imagery) were related positively with positive and negative post-task affect. The scale measuring the lack of awareness of surroundings (LA) was negatively correlated with post-task state-anxiety and the scale for emotional involvement (EI) was positively correlated to positive pre- and post-task affect (see Table 4).

**TABLE 4:** Pearson coefficients for associations between Transport Narrative questionnaire and respective subscales, Rate Arousal Sexual, Cortisol, Heart Rate, State Anxiety, and Positive and Negative Affect (df = 67).

	**1**	**2**	**3**	**4**	**5**	**6**	**7**	**8**	**9**	**10**	**11**	**12**	**13**	**14**	**15**	**16**	**17**	**18**	**19**	**20**
1. TT	–																			
2. CA	0.75**	–																		
3. FS	0.60**	0.32**	–																	
4. MI	0.83**	0.53**	0.37**	–																
5. LA	0.43**	0.35**	0.06	0.28*	–															
6. EI	0.64**	0.23*	0.15	0.50**	0.11	–														
7. RSA	0.59**	0.40**	0.46**	0.47**	0.33**	0.31**	–													
8. T−20	0.06	0.01	0.18	−0.01	0.03	−0.009	0.08	–												
9. T0	0.04	0.02	0.17	−0.02	0.10	−0.08	0.05	0.87**	–											
10. T+12	−0.09	−0.08	0.04	−0.13	0.13	−0.17	−0.03	0.67**	0.81**	–										
11. T+27	−0.03	−0.009	0.05	−0.09	0.11	−0.11	0.04	0.60**	0.71**	0.90**	–									
12. HR_B	−0.10	−0.11	0.12	−0.11	−0.25	−0.07	−0.08	0.21	0.28*	0.27*	0.20	–								
13. HR_T	0.01	−0.04	0.20	−0.01	−0.23	0.03	−0.001	0.25*	0.26*	0.21	0.16	0.93**	–							
14. HR_R	−0.01	−0.02	0.16	−0.07	−0.20	0.003	−0.01	0.25*	0.31*	0.24*	0.19	0.92**	0.91**	–						
15. S_P	−0.08	−0.22	−0.008	−0.09	−0.18	0.17	0.07	0.007	0.03	0.04	0.05	0.18	0.13	0.17	–					
16. S_PT	−0.13	−0.23	0.04	−0.13	−0.35**	0.09	−0.08	0.11	0.13	0.08	0.06	0.32**	0.38**	0.33**	0.72**	–				
17. PP_P	0.21	0.11	0.17	0.13	−0.05	0.28*	0.23	−0.001	−0.003	−0.04	−0.02	−0.001	0.01	−0.002	0.41**	0.09	–			
18. PP_PT	0.35**	0.28*	0.22	0.28*	−0.11	0.33**	0.24*	−0.08	−0.13	−0.23	−0.15	−0.15	−0.02	−0.04	0.21	0.16	0.63**	–		
19. PN_P	0.25	0.36	0.08	0.17	0.07	0.12	0.15	−0.10	−0.09	−0.13	−0.07	−0.16	−0.16	−0.12	−0.11	−0.25*	0.55**	0.62**	–	
20. PN_PT	0.37*	0.39**	0.23	0.29*	0.14	0.15	0.14	−0.10	−0.06	−0.09	−0.05	−0.21	−0.17	−0.13	−0.17	−0.27*	0.40**	0.64**	0.82**	–

TT, total transport narrative; CA, cognitive attention; FS, feeling of suspense; MI, mental imagery; LA, lack of awareness of surroundings; EI, emotional involvement; RSA, rate arousal sexual; T−20 to T+27, cortisol samples; HR_B, heart rate baseline; HR_T, heart rate task; HR_R, heart rate recovery; S_P, STAI state pre; S_PT, STAI state post; PP_P, PANAS positive pre; PP_PT, PANAS positive post, PN_P, PANAS negative pre; PN_PT, PANAS negative post; df, degrees of freedom. * *p* < 0.05; ** *p* < 0.01.

## Discussion

Existing work points to the efficacy of AD to guarantee immersion and emotional engagement (e.g., Fryer, 2013; Fryer and Freeman, 2013; Ramos, 2015, 2016). But evidence is still scarce, and more research is needed to explore its reception with different types of stimuli. The present study took on this challenge by testing the role of AD in both a sighted and a visually impaired audience’s engagement with porn films.

Results from the study confirmed our two main hypotheses, since no significant differences were found between groups or different versions of the clips. However, only a few of the expected patterns and correlations predicted in our two sub-hypotheses were confirmed. Overall, sighted and visually impaired participants reported to be moderately sexually aroused and immersed in the films, regardless of exposure to AV or AD porn. The mean for the three groups was around 4 on a 7-point Likert scale for both sexual arousal (AV = 3.65; AD = 3.88; ONCE = 4.15) and transportation (AV = 3.69; AD = 3.79; ONCE = 3.98). No difference was found either in physiological measures, with HR and cortisol secretion showing similar levels across the three groups. The lack of significant differences suggested that AD porn was capable of eliciting similar engagement and arousal to that of AV porn despite disability. It also supported previous results on the emotional response of AD reporting an effect as strong as that provided by original audiovisual films (cf. Fryer, 2013; Fryer and Freeman, 2013; Fryer and Jonathan, 2014; Walczak, 2017; Walczak and Fryer, 2018; Iturregui-Gallardo, 2019).

The only difference was found for the ONCE group’s levels of negative affect, which showed a significant decrease after exposure to AD porn. This result could be interpreted as a greater effect of the AD version on the self-perceptions of visually impaired participants’ as habitual consumers of AD content. This finding also aligns with previous evidence on the greater effect of AD over AV material for some constructs, such as the audience’s sense of presence or connection with the fictional environment (e.g., Fryer and Freeman, 2013). However, the effect was not corroborated by physiological data, since no differences were reported between the different versions regarding HR or cortisol secretion. The lack of agreement between self-report and physiological data in measuring emotional engagement is not uncommon (e.g., Richardson et al., 2020), since self-report data are subject to cognitive bias whereas physiological response is beyond conscious control.

A different explanation could be provided by the ONCE group’s attitude toward the experimental task. Visually impaired participants reported a higher level of negative affect in the pre-task questionnaire, which was later neutralized in the post-task, when their negative affect decreased after exposure to AD porn. A plausible explanation for this change is that visually impaired participants perceived the experiment as a negative situation: they were unfamiliar with the experimenters and probably feared the unknown. They knew the study involved envisioning films with explicit sexual content, and were probably anxious about having to experience porn in the presence of the experimenters. But once the session was settled and they were exposed to the films alone, they were able to relax and feel comfortable. In contrast, all sighted participants were students familiar with the experimenters, which could explain why they did not perceive the experimental situation as negative and why their affective state was not modified throughout the session. This explanation is, however, not supported by the ONCE group’s pre-task scores on the STAI-S, which showed no significant differences with the other groups, suggesting that visually impaired participants were not experiencing higher experimental anxiety than the rest. Moreover, their levels of trait anxiety were also significantly lower than the rest. The expected results on physiological measures predicted by hypothesis 3 were not corroborated. As predicted in hypotheses 1 and 2, cardiac and cortisol responses to the clips were similar in all the groups, but the experimental task did not elicit a higher cardiac response or a lower cortisol secretion when compared to the other phases of the session. The lack of cortisol response to sexual stimuli and even orgasm has been observed in previous studies reporting a similar cortisol response to both erotic and non-sexual stimuli (Heiman et al., 1991). However, there is also a possibility that the participants’ physiological response may have been influenced by their stress response. Theoretically, a physiological response to stress would inhibit sexual arousal (Hamilton et al., 2008: p. 1). In our study, both HR and cortisol responses were rather flat. If anything, the task vaguely decreased HR in all groups–with the ONCE group showing the smallest decrease–and faintly increased cortisol levels–with the group exposed to AV porn being the one showing the smallest increase. A plausible explanation for these tendencies may be the high levels of anxiety and negative affect reported by many participants. Their cardiac and cortisol responses seemed to indicate a decrease in their stress response by reducing both HR and cortisol, but the sexual arousal was not strong enough to suppress stress during intervention, resulting in a flat response. Besides, the possibility that the stimuli might not have been highly arousing should not be completely discarded, since according to the participants’ subjective perception, they were only moderately aroused. A higher arousal level may have been necessary to inhibit the stress response and produce an increase in cardiac response with the corresponding decrease in cortisol. Alternatively, the reported pattern in HR could also be interpreted as an orientation or attentional response caused by the novelty of the stimulus, as already indicated in classical studies (Bradley et al., 1993, 2001; Berntson et al., 1994). Image perception studies show a bradycardic pattern (decrease in HR) when people observe images of content that they consider interesting (typical of the orientation response), while they present an accelerative pattern when images are perceived as aversive (Bradley et al., 2001). In the case of AD porn, our participants did not see the images. And yet, since they knew a film was being played, there might have been an attentional response to the recorded stimulus, a reaction supported by evidence on the embodiment of narrative engagement which reports attentional focus to be related to lower HR levels (Sukalla et al., 2016). Moreover, the content of the clips was most likely perceived as non-aversive, pointing to a prospective bradycardic pattern. Still, this effect should be further tested by comparing our results with the response produced by interesting and aversive stimuli.

Regarding hypothesis 4, only a few correlations were found between the different measures. As previously mentioned, no correlation was reported between physiological indicators and self-report measures of sexual arousal and transportation. This result aligns with reported data on the lack of conclusiveness of physiological measures of emotion in AD studies (e.g., Ramos, 2016; Iturregui-Gallardo, 2019) and their disagreement with subjective measures in studies on sexual arousal (Heiman et al., 1991). In contrast, some correlations between self-report measures of sexual arousal, transportation and affect were reported in our study. A significant positive correlation was found between participants’ scores on the RSA and their scores on the Transport Narrative questionnaire for the total score and its five scales: cognitive attention (TCA), feeling of suspense (TFS), mental imagery (TMI), lack of awareness of surroundings (TLA), and emotional involvement (EI). This result suggests that the more immersed they were in the film, the more sexually aroused they felt.

Some correlations found between participants’ levels of self-reported sexual arousal and transportation and post-task affect pointed to a relationship between exposure to porn and perceived levels of sexual arousal and affect. For instance, positive post-task affect was positively correlated with scores on the RSA and the emotional involvement scale of the transportation questionnaire, that is, the more sexually aroused and emotionally involved in the film they felt, the higher their levels of positive affect. In addition, the scale measuring the lack of awareness of surroundings was negatively correlated with post-task anxiety, which suggested that the less aware of the experimental environment they became, the less anxious they felt. Finally, the total score on the transportation questionnaire and the scores for the cognitive attention and mental imagery scales were also positively correlated with both positive and negative post-task affect. Although further research would be needed to explain this result, the reported correlation could also be reflecting participants’ attentional response to the clips. Although evidence on the specific relationship between positive and negative affect and attentional scope and shift is not conclusive, results suggest that different affective states exert a different influence on scope of attention (e.g., Sadowski et al., 2020).

Results indicate that AD porn does provide a very similar experience to that of AV porn, but this study is exploratory and further research on the analysis of the AD reception of porn is needed. There are some obvious limitations to our research that should be addressed in future studies. Firstly, results suggest that the clips may not have been arousing enough to provoke the expected physiological response. A way to guarantee a stronger sexual response would be to use scenes adapted to participants’ individual sexual preferences, since there is evidence suggesting the importance of using stimuli that align with participants’ sexual orientation in sex research protocols (Pulverman et al., 2015). There is also the possibility that 12 min was not long enough to increase sexual arousal in the experimental situation, especially given many participants’ high levels of pre-task trait anxiety and negative affect. Even if similar length stimuli have proven successful in previous studies on sexual arousal (Hamilton et al., 2008; Rellini et al., 2009), there are also experimental designs with different films of around 20 min each (Heiman et al., 1991; Exton et al., 2000). Moreover, they all included a non-sexual documentary as control. The use of such a control would have been useful to determine whether the response was due to the fact that the stimuli were not arousing enough.

Secondly, another limitation relates to the physiological measures employed. Cortisol has been used before to measure sexual response but results on cortisol response show no consistent pattern. Cortisol in women may show no change, decrease or even increase, depending on their levels of anxiety, histories of medical or psychological disorders, being on the pill or even the phase of their menstrual cycle. No cases of medical or psychological disorders were found in our sample. As previously mentioned, participants were also asked for their phase of the menstrual cycle, but since most of them admitted not being sure of their answers, the variable was not included in the study. Women on hormonal contraceptives were also included, since there is evidence pointing to no differences with women in their follicular phase in their salivary cortisol responses to sexual stimuli (Hamilton et al., 2008). Phases of the menstrual cycle should, nevertheless, be controlled for in future studies to discard their potential effect.

Likewise, HR measures represent the effect of the relative contributions of an excitatory system (SNS) and an inhibitory system (the PSNS), but the inclusion of some additional sympathetic and parasympathetic indices related to cardiovascular activity would be necessary to better characterize the autonomic activity supporting cardiac response. The use of HRV in future studies would be advisable to determine whether the response is due to film exposure or to an increase in participants’ parasympathetic activity.

Finally, limitations on our sample should also be acknowledged. In all fairness, 69 participants may not be a high enough number to detect definite trends but is a reasonable number for AD studies on visually impaired participants, a population not at all easy to access. However, even if results agree with existing studies with similar populations, the study should be replicated with more participants. The difficulties to recruit visually impaired participants also explain another limitation of the sample related to differences in group profiles. Visually impaired participants were significantly older and showed lower trait anxiety levels than sighted participants. Differences are also to be acknowledged within the ONCE group, where participants had different economic and academic backgrounds, and even different levels of visual disability. Heterogeneity is, nevertheless, the reality of the visually impaired population, a highly varied group who cannot be easily homogenized.

## Conclusion

Notwithstanding the limitations mentioned above, the present paper offers the first study on the emotional reception of AD porn. Results from the study point to the efficacy of AD for the blind in providing sighted and visually impaired audiences with a similar experience to that offered by original AV porn scenes. Results on physiological and self-report measures revealed no significant differences between groups or different versions of the clips. The analysis of cortisol reactivity to porn disclosed no significant differences between the groups, but showed a higher percentage of non-responders than responders in the three groups, the highest being found in the ONCE group. As for participants’ HR response to the clips, no significant differences were found either across the groups, with the highest HR levels being registered in the baseline phase. In contrast, self-report measures pointed to significant between-group differences in negative affect. The ONCE group showed the highest pre-task levels of negative affect and was the only group that showed a decrease in negative affect after exposure to AD porn. Both sighted and visually impaired participants reported to be moderately aroused and immersed or transported by the films, regardless of exposure to AV or AD porn. Moreover, the correlations found between participants’ levels of self-reported sexual arousal, transportation and post-task affect pointed to a positive relationship between exposure to porn and perceived levels of sexual arousal and affect.

The present study is exploratory but provides valid, initial groundwork for further research. Porn has already started the road to inclusiveness and accessibility, but has a lengthy path ahead. There are plenty of cases showing that porn is still not accessible to all. The lawsuit filed at the beginning of 2020 by a hearing-impaired man in Brooklyn against Pornhub and other pornographic sites for the lack of subtitles on the platform’s videos recently exposed the poor accessibility of pornography. The litigation disclosed that hearing impaired users had been prevented from getting full and equal enjoyment when watching porn (Katersky and Torres, 2020). And visually impaired users are not an exception either. But what are the existing solutions to make porn accessible? For blind and partially sighted people, AD is the bedrock of greater accessibility and its usability is increasingly attracting interest. The continuous surge of new forms of visual art requires the development of new AD styles adapted to the new needs. For the present study, the AD was recorded in a classical style, but evidence on emerging styles, such as radio drama or immersive soundscape with AST (see case studies from The Immersive Accessibility Project (ImAc) at the Royal National Institute of Blind People webpage), points to improved effects on immersion and emotional engagement.

If porn is to be made accessible to the blind population, research can help us know what should and should not be done to assure an equal experience for sighted and visually impaired consumers. Our study has suggested that AD can guarantee such a similar experience in terms of physiological reactivity and subjective sexual arousal, but there are many other aspects of this experience that remain to be explored. Individual preferences, for instance, still need to be accommodated and accounted for in research designs. While some homosexuals may prefer gay or lesbian porn, others may equally enjoy straight or heterosexual porn. Some blind consumers may think that music or narrative adds to the scene, but others may find them distracting. Surely, the experience of visually impaired consumers can be improved if they are allowed to find exactly what they want to watch. Wholly accessible porn should not only be within easy reach of hearing and visually impaired users, but also be equally rewarding and enjoyable for all, regardless of their abilities. So far, AD seems enough to guarantee a similar experience, but much more can be done to guarantee immersion and enjoyment. A more dramatized style and immersive soundscape can surely help, but further research is needed to test their impact on porn reception. Getting sexually aroused without seeing should no longer be in question; blind and partially sighted users of porn have the right “to feel” the same as sighted ones. Inclusive and accessible porn should not be the desideratum of a few, but rather a right made true for all.

## Data Availability Statement

The raw data supporting the conclusions of this article will be made available by the authors, without undue reservation.

## Ethics Statement

The studies involving human participants were reviewed and approved by University of Murcia Ethics Research Committee. The patients/participants provided their written informed consent to participate in this study.

## Author Contributions

All authors designed the study, analyzed the cortisol data, contributed toward drafting and revising the manuscript, and agreed to be accountable for all aspects of the work. AR and LE conducted the statistical analyses. MR and AR collected the data.

## Conflict of Interest

The authors declare that the research was conducted in the absence of any commercial or financial relationships that could be construed as a potential conflict of interest.

## Footnotes

1 The criterion for inclusion in the ONCE is having, at least, one of the following visual conditions in both eyes, and a reliable prognosis of no visual improvement: (a) visual acuity equal to or less than 0.1 obtained with the best possible optical correction, (b) visual field reduced to 10 degrees or less.
